# Psychological intervention for universal prevention of antenatal and postnatal depression among pregnant women: protocol for a systematic review and meta-analysis

**DOI:** 10.1186/s13643-019-1238-7

**Published:** 2019-12-01

**Authors:** Naonori Yasuma, Zui Narita, Natsu Sasaki, Erika Obikane, Junpei Sekiya, Takuma Inagawa, Aiichiro Nakajima, Yuji Yamada, Ryuichi Yamazaki, Asami Matsunaga, Tomomi Saito, Kazuhiro Watanabe, Kotaro Imamura, Norito Kawakami, Daisuke Nishi

**Affiliations:** 1 Department of Mental Health, Graduate School of Medicine, The University of Tokyo, 7-3-1 Hongo, Bunkyo-ku, Tokyo, 1130033 Japan; 2 Department of Psychiatry and Behavioral Sciences, The Johns Hopkins University School of Medicine, Baltimore, USA; 3 Department of Psychiatry, Negishi Hospital, Fuchu, Tokyo, Japan; 4 Department of Psychiatry, National Center of Neurology and Psychiatry Hospital, Kodaira, Tokyo, Japan; 5 Department of Psychiatry, The Jikei University School of Medicine, Minato-ku, Tokyo, Japan; 6 Department of Community Mental Health and Law, National Institute of Mental Health, National Center of Neurology and Psychiatry, Kodaira, Tokyo, Japan; 7 Department of Obstetrics and Gynecology, School of Medicine, Juntendo University, Bunkyo-ku, Tokyo, Japan

**Keywords:** Antenatal depression, Postnatal depression, Psychological intervention, Universal prevention

## Abstract

**Background:**

Prevention of antenatal and postnatal depression is crucial, given its high prevalence and severe consequences. Although several systematic reviews and meta-analyses have examined the effects of psychological interventions on the population at risk for perinatal depression, few studies have focused on universal prevention and none have focused specifically on universal prevention in pregnancy. The aim of this study is to examine the effects of psychological interventions with a universal prevention focus on perinatal depression during pregnancy by performing a systematic review and meta-analysis based on both the latest articles and a broader literature search.

**Methods:**

The literature search will be conducted using the Cochrane Controlled Register of Trials (CENTRAL), Embase, PubMed and PsycINFO, from inception onwards. Randomized controlled trials that examined the association between psychological interventions and universal prevention of antenatal and postnatal depression among pregnant women will be included. Study selection, data collection, quality assessment, and statistical syntheses will be conducted following a priori defined methods in the protocol.

**Discussion:**

The findings of this systematic review and meta-analysis will have both clinical and political importance in the context of perinatal mental health. In addition, this study will promote future studies and clarify the direction of research on universal prevention of perinatal depression.

**Systematic review registration:**

PROSPERO CRD42019118041

## Background

Prevention of antenatal and postnatal depression has always been a critical issue in perinatal mental health [1]. The prevalence of antenatal and postnatal depression is approximately 10% each [2]. Antenatal depression has been associated with inconsistent antenatal examinations, inadequate diet, smoking, alcohol consumption, substance abuse, and the risk of self-harm or suicide [3–7]. In addition, it also has been associated with slow fetal growth and paternal depression [8, 9]. Similarly, postnatal depression has been related to suicide as well as to negative outcomes, such as child neglect, child abuse, and infanticide [10, 11]. Therefore, the prevention of antenatal and postnatal depression is an urgent matter.

In a previous systematic review and meta-analysis, psychological interventions were recommended as the most effective approach to prevent antenatal and postnatal depression, especially among individuals with risk factors, such as a history of depression, lack of social support, and unwanted pregnancies [12, 13]. Among psychological interventions, cognitive behavioral therapy (CBT) and interpersonal therapy (IPT) have been widely studied and found to be highly effective [14, 15]. Other interventions, such as physical activity, infant sleep education, in-hospital perinatal education, and peer counseling, might also be effective, although further studies with larger sample sizes are warranted [12]. Similarly, dietary supplements such as selenium and vitamin D have been shown to be partially effective, although more research is needed to explore these interventions [12].

Although several systematic reviews and meta-analyses have examined the effects of psychological interventions on the population at risk for perinatal depression, only a few studies have focused on universal prevention which refers to approaches designed for an entire population regardless of individual risk factors and none focused specifically on universal prevention from pregnancy [16, 17]. Considering the magnitude of psychological distress and burden of the disease, depression during pregnancy is itself in need of prevention [18]. Moreover, it may not be feasible to identify all high-risk pregnant women with screening tests [19]. A large number of pregnant women have risk factors, such as lack of social support and unwanted pregnancy, while other pregnant women develop depression without these risk factors [20]. Furthermore, compared to the general female population, pregnant women experience various physical and environmental changes in the postnatal period, such as sudden hormonal imbalance and the baby crying at night [21, 22]. Therefore, psychological interventions are needed that specifically target the perinatal condition and additional systematic reviews and meta-analyses of psychological interventions for universal prevention among pregnant women are required.

Accordingly, the objective of this study is to evaluate the effects of psychological interventions on antenatal and postnatal depression during pregnancy by performing a systematic review and meta-analysis based on the latest articles and a broader literature search.

## Methods

### Study design

We registered this systematic review protocol in the PROSPERO international prospective register of systematic reviews (registration number, CRD42019118041). The protocol was prepared using the 2015 statement of Preferred Reporting Items for Systematic Reviews and Meta-Analysis Protocols (PRISMA-P) [23]. The final review will be reported using the Preferred Reporting Items for Systematic Reviews and Meta-Analysis (PRISMA) statement [24].

### Search strategy

We will conduct literature searches using the following electronic bibliographic databases from inception onwards: Cochrane Controlled Register of Trials (CENTRAL), Embase, PubMed, and PsycINFO. Our search terms consist of “perinatal” and “preventing depression”. For “perinatal”, the search term was based on the study of the US Preventive Services Task Force (USPSTF) [12]. For “preventing depression”, we used the same expression as the previous study, which was a meta-analysis of preventing depression [25]: (pregnan* OR antenatal* OR ante-natal* OR antepartum* OR ante-partum* OR prenatal* OR pre-natal* OR mother* OR (expectant mother*)) AND (("depressive disorder"[MeSH Terms] OR ("depressive"[All Fields] AND "disorder"[All Fields]) OR "depressive disorder"[All Fields] OR "depression"[All Fields] OR "depression"[MeSH Terms]) OR “depressive” [All Fields]) AND (("prevention and control"[Subheading] OR ("prevention"[All Fields] AND "control"[All Fields]) OR "prevention and control"[All Fields] OR "prevention"[All Fields]) OR “preventive”[All Fields]). For grey literature, we will scan the reference lists of included studies and previous reviews.

### Eligibility criteria

Individual or cluster randomized controlled trials will be included. We will exclude quasi-randomized trials and crossover studies. Original articles written in English will be included. The following participants, interventions, comparisons, and outcomes (PICO) of the studies in this systematic review and meta-analysis will be included: (P) All adult pregnant women (over 18 years old) regardless of risk factors for depression and without any racial restrictions, (I) psychological interventions such as cognitive behavioral therapy (CBT) and inter personal therapy (IPT), (C) usual care which did not include any psychological intervention, (O) antenatal and postnatal depression diagnosed by interview with medical professionals or measured by using a self-reported psychological inventory whose reliability and validity has been confirmed. There are no exclusion criteria. If the study has more than two arms, we will compare each psychological intervention with usual care.

### Study selection

NY will exclude duplicate studies before the screening. We will divide 12 investigators (NY, DN, EO, NS, ZN, JS, TI, AI, YY, RY, AM, TS) into 6 groups of 2 people each. NY will screen all the manuscripts from title and abstract and then each group of two people will be in charge of one sixth of the whole manuscripts and independently perform a first screening from the title and abstract. To improve the accuracy of the first screening, we will proceed as follows. First, the first author (NY) will prepare a manual on the inclusion and exclusion criteria of this study and provide it to all members. Second, at least one experienced systematic reviewer will be assigned to each group. In this stage, Kappa statistics of each two investigators will be calculated to assess if their ratings and agreement are reliable. Third, the study selected by at least one reviewer will be judged in the full-text screening. Subsequently, NY and ZN will independently evaluate the full-text eligibility, after which the senior reviewer (DN) will reconcile any disagreements.

### Data extraction

NY and ZN will independently extract the following relevant information from included studies: author, year of publication, country, number of participants, details of the intervention, number of sessions, time of one session, control condition, duration of follow-up, and measurement tools. The senior reviewer (DN) will reconcile any disagreements. If the study only shows the presence of perinatal depression by using the cut-off value of the self-administered questionnaire, we will ask the author for additional information about the continuous outcomes. If this method is not successful or feasible, we will exclude these studies from the analysis. If perinatal depression was diagnosed by a medical professional, we will report the study narratively.

### Quality assessment and risk of bias

NY and ZN will independently conduct a quality assessment using the Cochrane Collaboration’s risk of bias tool, which assesses potential biases in the following domains: sequence generation, allocation concealment, blinding of participants and personnel, blinding of outcome assessors, incomplete outcome data, selective outcome reporting, and other sources of bias [26]. The senior reviewer (DN) will reconcile any disagreements.

### Statistical analyses

Meta-analysis will not be performed if only one or two studies fulfill the eligibility criteria. In this case, the findings will be summarized in a narrative format. We also will conduct narrative synthesis if conducting meta-analysis is not appropriate due to a large heterogeneity. A pooled effect size will be synthesized into standardized mean differences (SMD) in the continuous outcomes between groups (i.e., intervention and control) calculated by the number of participants, mean difference, and standard deviation. A random effect model will be conducted using Review manager 5.3 (Cochrane Collaboration software). Heterogeneity will be assessed using the Cochrane’s *Q* test and *I*^2^ test. *I*^2^ values of 25%, 50%, and 75% indicate low, medium, and high heterogeneity, respectively [26]. If there are 10 or more studies in the meta-analysis, we will examine publication bias by visual inspection of funnel plots. If data are available, subgroup analyses will be conducted for diverse types of psychological interventions, follow-up duration, antenatal or postnatal, country, and primiparous or multiparous.

## Discussion

Our study will systematically review and analyze the evidence for the effect of psychological interventions as a universal approach to the prevention of antenatal and postnatal depression among pregnant women.

The strength of this study is that it will examine a wider range of electronic bibliographic databases, including CENTRAL and Embase and will be based on the latest articles. In addition, considering the high prevalence and severe consequences of antenatal and postnatal depression, the findings obtained in the current study may be beneficial in the context of perinatal mental health, both clinically and politically. Furthermore, this study will promote future studies and clarify the direction of research on universal prevention of perinatal depression.

However, the proposed systematic review and meta-analysis have some potential limitations. There may be a language bias, as we will include only studies published in English, which may result in the exclusion of some relevant studies in other languages. Another limitation is that the assessment of antenatal and postnatal depression using different methods may cause heterogeneity across studies.

## Data Availability

Not applicable

## Abbreviations

PRISMA: Preferred Reporting Items for Systematic Reviews and Meta-Analysis
PRISMA-P: Preferred reporting items for systematic review and meta-analysis protocols
